# Experimental Study on the Fracture Parameters of Concrete

**DOI:** 10.3390/ma14010129

**Published:** 2020-12-30

**Authors:** Zhanqiao Wang, Jin Gou, Danying Gao

**Affiliations:** 1 School of Water Conservancy Engineering, Zhengzhou University, Zhengzhou 450001, China; wzqzzu@zzu.edu.cn; 2 School of Civil Engineering, Zhengzhou University, Zhengzhou 450002, China; gdy@zzu.edu.cn; 3 College of Civil Engineering, Henan University of Engineering, Zhengzhou 451191, China

**Keywords:** steel fiber, three-point bending, fracture parameters, gain ratio

## Abstract

This study aimed to determine the influence of the volume fraction of steel fibers on the fracture parameters of concrete. Fifty notched steel-fiber-reinforced concrete (SFRC) beams and ordinary concrete beams with 100 mm × 100 mm × 515 mm were cast and tested via a three-point bending test. Among them, the type of steel fiber was the milling type (MF), and the volume fraction of steel fiber added was 0%, 0.5%, 1%, 1.5% and 2%, respectively. The effects of the steel fiber volume fraction (V_F_) on the critical stress intensity factor (K_IC_), fracture energy (G_F_), the deflection at failure(δ_0_), the critical crack mouth opening displacement (CMOD_C_) and the critical crack tip opening displacement (CTOD_C_) were studied. Through the analysis of test phenomena and test data such as the load-deflection (P-δ) curve, load-crack mouth opening displacement (P-CMOD) curve and load-crack tip opening displacement (P-CTOD) curve, the following conclusions are drawn: with the increase of the steel fiber volume fraction, some fracture parameters increase gradually and maintain a certain linear growth. The gain ratio of the fracture parameters increases significantly, and the gain effect is obvious. Through this law of growth, the experimental statistical formulas of fracture energy and the critical stress intensity factor are summarized.

## 1. Introduction

Steel-fiber-reinforced concrete (SFRC) has been emerging as a widely used construction material that is suitable for many structural elements [1,2,3]. SFRC has been applied in industrial and airport pavements, projected concrete members and precast concrete elements, reducing the concrete’s thickness [3,4].

SFRC is a composite material produced by the reinforcement of a cementitious matrix in which short-length steel fibers are randomly distributed. Compared with ordinary concrete, the incorporation of steel fibers is receiving growing attention, as it offers many advantages, such as tensile strengths, toughness and durability [5,6,7,8,9,10]. SFRC can effectively make up for the defects of concrete, which is easy to crack [3], improve the mechanical properties of roads and promote the development of the road construction industry.

The remarkable feature of steel fiber is that it hinders the development of macro cracks, delays the propagation of micro cracks to macro cracks and improves the ductility of micro cracks [1,8,9,10,11,12,13]. This performance enhancement under tension and bending is mainly due to the better post-cracking behavior of fiber-bridging cracks, which leads to a much higher energy absorption capacity than that of ordinary concrete [1,11]. Many researchers have shown that including steel fibers provides a higher flexural strength, deflection capacity, and post-peak ductility than plain concrete, and this strength and ductility increases with the increase in the fiber content [2,14]. Through these experiments, it was also proved that adding steel fiber and increasing the amount of fiber can significantly improve the fracture toughness and energy absorption capacity of concrete.

Fracture performance is important for the safety and durability of concrete structures. A basic ingredient for measuring the fracture process in quasi-brittle materials is the fracture energy (G_F_) [1]. F. Bencardino et al. [1] reported that adding fibers to concrete has a major influence on the fracture property and ductility of the FRC. According to Doo-Yeol Yoo et al. [2], a steel fiber content of more than 1.0% can significantly improve the flexural strength, flexural capacity and fracture energy of concrete, and these factors will also increase with the increase of the steel fiber content. M.T. Kazemi et al. [15] studied the fracture properties of SFHSC and found that the fracture properties of SFHSC increased with the increase of the steel fiber content. The above studies show that the content of steel fibers has a significant effect on the properties of concrete.

In the literature, different methods have been proposed for estimating the fracture parameters experimentally: the direct uniaxial tension test, the wedge splitting test and bending tests [1]. In order to ensure the uniform mixing of steel fibers with different volume fractions, it is necessary to increase water consumption in different degrees. In order to eliminate the influence of matrix concrete strength variation on the test results and objectively reflect the gain effect of steel fiber on the mechanical properties of SFRC, the concept of gain ratio is introduced, based on the study of SFRC fracture parameters. The gain ratio is the ratio between the fracture parameters of SFRC and the fracture parameters of conventional concrete specimens poured with the corresponding mixture proportion. A three-point bending test was used to evaluate the effect of fiber content on the fracture parameters of SFRC, and the effect was explained by experimental phenomena, test process curve and calculated gain ratio. Two test statistical equations of the volume fraction of steel fibers and fracture energy (V_F_-G_F_), as well as the volume fraction of steel fibers and the critical stress intensity factor (V_F_-K_IC_) are established, where R^2^ = 0.97 and 0.99, respectively. This study can further promote the understanding and application of steel fiber reinforced concrete.

## 2. Summary of Test

### 2.1. Materials

In this test, according to CECS13:89 [16], PO42.5 Portland cement was selected, mid-coarse river sand with a modulus of fineness of 3.39 and limestone with a maximum particle size of 20 mm and continual size distribution. FDN-1 high efficiency water reducing agent with 1% cement dosage was also used. The milling steel fiber with a length-diameter ratio of 34.42 was adopted. Their tensile strength is higher than that of 700 MPa. The milling steel fiber fraction consisted of 0, 0.5%, 1.0%, 1.5% and 2%, respectively [17].

### 2.2. Mix Design

According to CECS13:89 [16], the reference concrete mix proportion is m(water): m (cement): m(sand): m(stone): m(FDN-1) = 164: 341.7: 661.1: 1283.3: 1.64. In order to ensure the uniform mixing of steel fiber, for every 0.5% increase in V_F_, the water dosage increased by 8 kg. In order to eliminate the influence of the strength variation of the matrix concrete on the test results, at the time of pouring SFRC, the ordinary concrete (OC) specimens with the same mix proportion as SFRC but without steel fiber were used as control specimens. The mix proportion of SFRC specimen and control group concrete is shown in Table 1.

### 2.3. Specimen Preparation Procedure

**Figure 1:**
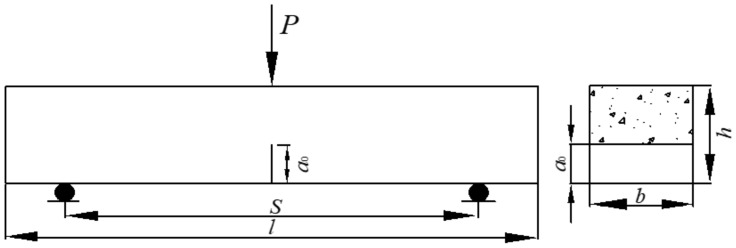
Sketch of the specimen’s shape and dimension.

Notched three-point bending beams as shown in Figure 1. The specimens were 100 mm wide, 100 mm high and 515 mm long, the span S was 400 mm and the relative notch depth of the specimens, a_0_/l, was 0.4. The design strength grade of SFRC was FC30.

In order to improve the uniformity of the mixture of SFRC and the control group’s ordinary concrete (OC) specimens and to avoid segregation, a superplasticizer was used and the mixing time was prolonged. In order to make the fiber well distributed and avoid agglomeration, the slow dispersion method was adopted when adding steel fiber, and a forced mixer was used. The concrete of SFRC and its control group was mixed by forced mixer, formed by a shaking table, cured in the standard curing room (temperature 20 ± 3 °C, relative humidity not less than 95%) for 28 days, and then naturally cured and processed to the test room after maintenance. The experimental age was between 50 days and 70 days.

### 2.4. The Three-Point Bending Test

The three-point bending test was carried out on a hydraulic press testing machine with a maximum capacity of 2000 kN. In order to ensure the continuous stability of the test process, the disc spring reinforcement device of the testing machine was used to strengthen the testing machine. The load sensor was used to measure the load of the notched three-point bending beam, and the measuring range of the sensor was 0–30 kN. In order to eliminate the influence of bearing deformation on the deflection test results, the deflection test beam was arranged directly above the support point of the three-point bending specimen, and the mid-span deflection is measured with a percentile meter. The CMOD clip extensometer was arranged at the bottom of the beam with a 3-mm thick alloy aluminum knife edge. The CTOD clip extensometer was fixed at the tip of the crack on the side of the beam with a knife edge.

**Figure 2:**
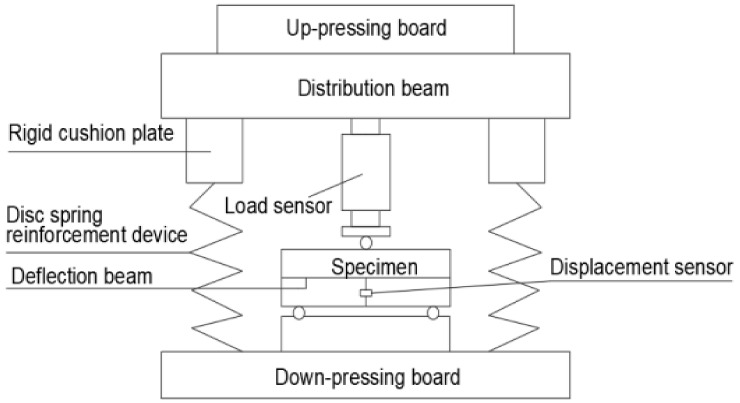
Sketch of the loading set-up for the three-point bending test.

The continuous loading mode was adopted in the test, and the computer data acquisition system was used to realize the automatic data acquisition. The acquisition rate was different according to the type of the component, i.e., concrete component 2 times/s, SFRC component 1 time/s. The load deflection (P-δ) curve, load-CMOD (P-CMOD) curve and load-CTOD (P-CTOD) curve can be observed simultaneously. According to these test curves and the observation of test phenomena during the test, the fracture properties of SFRC can be analyzed qualitatively and quantitatively. The experimental loading and measurement device can be seen in Figure 2.

### 2.5. Calculation Formula of the Fracture Parameters

Calculation Formula of the Critical Stress Intensity Factor (K_IC_)

In China, the K_IC_ calculation formula in ASTM E399-1972 is often used. The calculation formula of K_IC_ is as follows:

(1) $$K_{\mathrm{IC}}=\frac{P_{\max}S}{{\mathrm{bh}}^{1.5}}f\left(\frac{a}{h}\right)$$

(2) $$f\left(\frac{a}{h}\right)=2.9{\left(\frac{a}{h}\right)}^{0.5}-4.6{\left(\frac{a}{h}\right)}^{1.5}+21.8{\left(\frac{a}{h}\right)}^{2.5}-37.6{\left(\frac{a}{h}\right)}^{3.5}+38.7{\left(\frac{a}{h}\right)}^{4.5}$$

where P_max_ is the measured maximum load (N), S is the span of beam (mm), b is the width of the sample(mm), h is the height of the sample (mm) and a is the actual crack length (mm). The virtual crack length is used in this paper.

The Calculation Formula of Fracture Energy (G_F_) [12,18]

(3) $$G_{F}=\frac{W_{0}+{\mathrm{mg}\delta }_{0}}{b\left(h-a_{0}\right)}$$

where W_0_ is the area under the load-deflection curve (N·m), m is the total mass of specimens between supports (kg), g is the gravitational constant (N/kg), δ_0_ is the deflection at failure (mm), h is the height of the sample (mm), a_0_ is the depth of the notch (mm) and b is the width of the sample (mm).

## 3. Test Results and Analysis

The testing results of the fracture parameters of SFRC and OC are shown in Table 2. All test results were reported as the average value of tested specimens.

In the correlation analysis of this paper, the gain ratio is used to reflect the reinforcement effect of steel fiber, which is defined as: gain ratio = mechanical performance index of SFRC/mechanical performance index of the corresponding control group’s concrete specimen. For example: fracture toughness gain ratio = fracture toughness of SFRC specimens/fracture toughness of OC specimens of the same mix proportion.

### 3.1. Fracture Toughness

**Figure 3:**
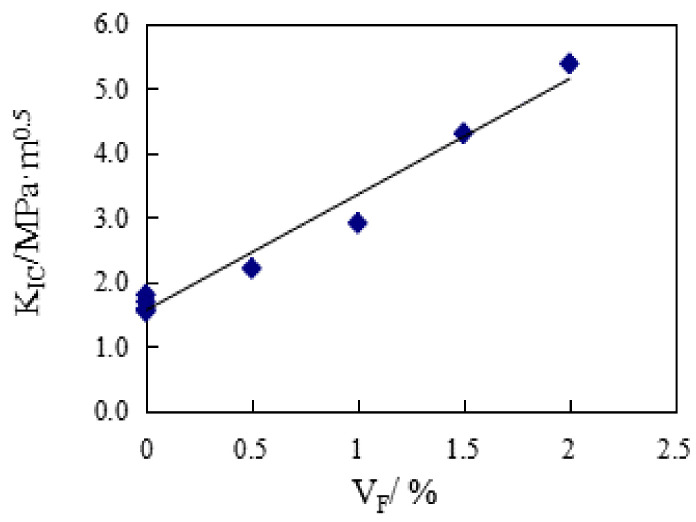
The effect of V_F_ on K_IC_.

The effect of V_F_ on K_IC_ is shown in Figure 3. It can be seen, when compared with the reference group concrete, that the addition of steel fiber significantly improves the fracture toughness of SFRC. And with the increase of V_F_, the K_IC_ of SFRC specimens show a good increasing trend. For every 0.5% increase in the fiber fraction, the average increase in K_IC_ is 33.7%. The K_IC_ gain ratio of V_F_ to SFRC can be seen in Table 2: with the addition of steel fiber, the K_IC_ gain ratio of SFRC specimens is greater than 1, and with the increase of V_F_, the gain ratio shows a good increasing trend, the minimum gain ratio is 1.43, the maximum gain ratio is 3.45 and the average gain ratio is 2.275. Therefore, it can be considered that the addition of steel fiber improves the K_IC_ of concrete.

According to the test results of KIC in this paper, the statistical analysis shows that:

(4) $$\;K_{IC}=1.7925V_{F}+1.5634$$

where R^2^ = 0.9748. It can be seen that the increase of K_IC_ is mainly affected by V_F_.

**Figure 4:**
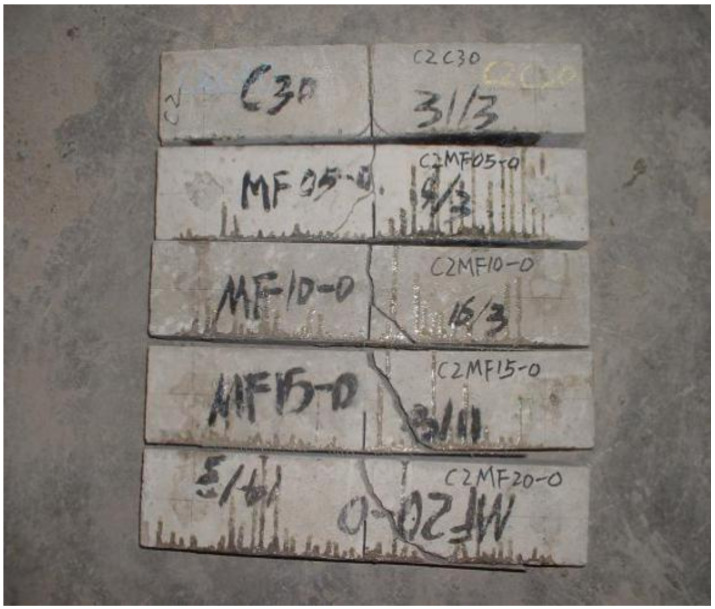
The failure of OC.

**Figure 5:**
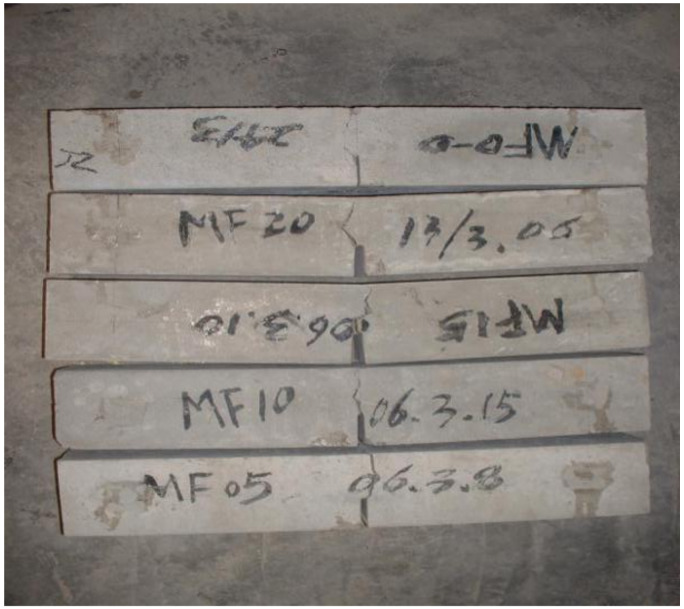
The failure of SFRC.

The failure patterns of three-point bending specimens with different V_F_ are compared, as shown in Figure 4 and Figure 5.

It can be seen from the test process that the SFRC specimen can be clearly heard the sound of the fiber pulling out or breaking in the test. E.K. Tschegg [19] also obtained similar results. With the addition of steel fiber, the crack development path becomes relatively tortuous, from the single crack propagation of the reference group to the multi-point cracking of more than one crack, and with the increase of V_F_, the crack propagation path becomes more tortuous. Except for a small number of SFRC specimens with small content, some specimens were split in half, but most of the them were not completely split, which kept the integrity to a certain extent. On the other hand, the K_IC_ of the ordinary concrete of the reference group and control group was small, and the bearing capacity of the specimens decreased rapidly after the test load reached the peak, which led to most of the specimens splitting into two parts. It can be judged that the addition of steel fiber greatly improves the fracture toughness of SFRC.

### 3.2. Fracture Energy

**Figure 6:**
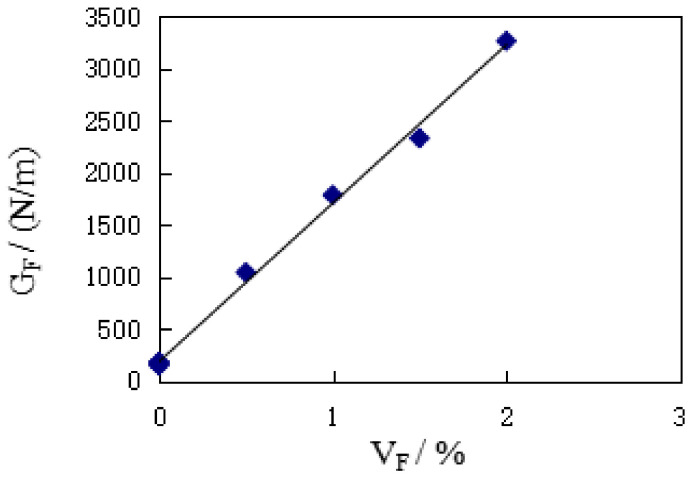
The effect of V_F_ on G_F._

Figure 6 shows the effect of V_F_ on the G_F_ of SFRC. It can be seen, compared with the reference group concrete, that the addition of steel fiber greatly improves the fracture energy of concrete matrix, and with the increase of V_F_, G_F_ keeps an obvious linear increasing trend. For every 0.5 percentage point increase in V_F_, G_F_ increases between 30.53% and 71.14%, with an average increase of 47.22%. It can be seen from Table 2 that, compared with the control group concrete, the G_F_ gain ratio of SFRC specimens is between 5.88 and 20.52, and that the average gain ratio is 12.88. It can thus be seen that the addition of steel fiber greatly improves the energy absorption capacity of the concrete matrix.

According to the test results of K_IC_ in this paper, the statistical analysis shows that:

(5) $$G_{F}=1520.6V_{F}+186.18$$

where R^2^ = 0.9966. It can be seen that the increase of G_F_ is mainly affected by V_F_.

**Figure 7:**
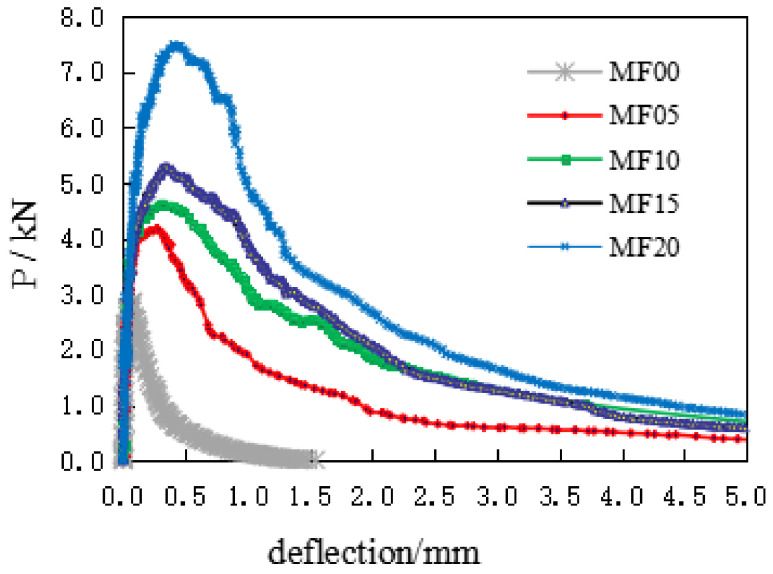
The typical load-deflection curves.

Figure 7 shows the comparison of typical load deflection (P-δ) curves of a group of experiments under different V_F_ conditions.

It can be seen from the figure that the deflection of concrete specimen is almost linear elastic before reaching the peak load. This result is consistent with the test results of Doo-Yeol Yoo [2]. As the load continues to increase, the deformation of the matrix concrete reaches the initial crack strain, the concrete matrix shows micro-cracks, and P-δ curves begin to show a non-linear growth. With the emergence of macroscopic cracks, the matrix concrete cracks continue to expand, and the steel fiber reinforced concrete is in the elastic-plastic stage. At this time, in the process of gradually pulling out, the steel fiber connects the concrete across the cracks, so that the steel-fiber-reinforced concrete shows better ductility. With the increase of V_F_, the number of fibers across the cracks increases accordingly, and the ability to transfer stress is also relatively improved. The microcracks in the fracture process zone develop fully, and the absorbed external load energy increases accordingly, which is characterized by the increase of the peak load and ultimate deflection of the specimen. The test curve becomes fuller and the improvement of fracture performance is more sufficient.

### 3.3. Crack Opening Displacement

From the test data analysis in Table 2, it can be seen that, compared with the reference group concrete specimens, the addition of steel fiber increases the CMOD_C_ and CTOD_C_ of matrix concrete, and that with the increase of V_F_, CMOD_C_ increases linearly, and the deflection corresponding to CMOD_C_ also increases obviously.

**Figure 8:**
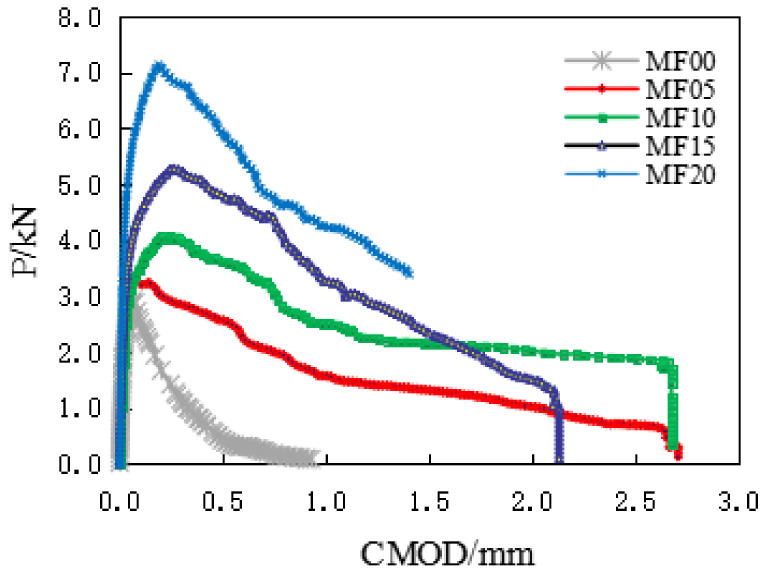
The typical load crack mouth opening displacement (CMOD) curves.

**Figure 9:**
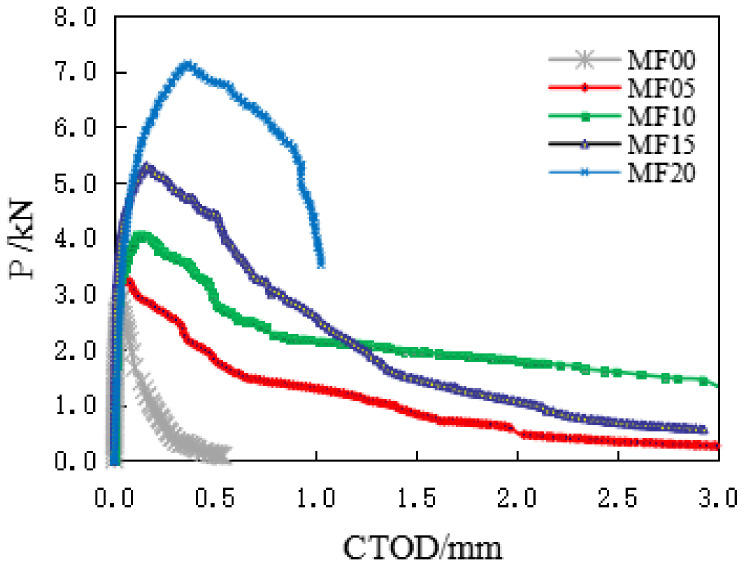
The typical load crack tip opening displacement (CTOD) curves.

The typical load-CMOD diagram and load-CTOD diagram of a group of SFRC specimens recorded during the bending test is shown in Figure 8 and Figure 9. It can be seen from the figure that the crack opening displacement of the ordinary concrete specimen is almost linear elastic before reaching the peak load, and the crack opening displacement of the fiber-reinforced concrete specimen also shows linear elasticity at the initial stage of applying the load. It gradually enters the plastic stage with the continuous increase of the load, which is an extensive cracking process, obviously different from the ordinary concrete specimen.

With the increase of the load, the steel fiber is gradually pulled out, and it connects the concrete across the cracks. The high content of steel fiber increases the bridging effect, which makes the steel-fiber-reinforced concrete show better ductility. In the test, the peak load and critical crack opening displacement of concrete specimens increased.

## 4. Conclusions

Through the analysis of the fracture behavior of concrete notched three-point bending beam specimens of SFRC and their control group, the following conclusions can be drawn:

1. The addition of steel fiber to concrete can obviously improve the fracture toughness of concrete. With the increase of V_F_, the K_IC_ of SFRC specimens showed a good increasing trend. For every 0.5% increase in the fiber volume rate, the average increase of K_IC_ is 33.7%, and the average gain ratio is 2.275.
2. Adding steel fiber to concrete can obviously improve the fracture energy of concrete. With the increase of V_F_, the G_F_ and G_F_ gain ratio of SFRC specimens showed a good increasing trend. In the range of V_F_, for every 0.5% increase of V_F_, the average gain ratio of G_F_ is 47.22%, the average gain ratio is between 5.88 and 20.52, and the average gain ratio is 12.88.
3. With the addition of steel fiber, the peak loads, the deflection at failure and the critical crack opening displacement of concrete specimens increased.
4. Two test statistical equations of V_F_-G_F_ and V_F_-K_IC_ are established, where R^2^ = 0.97 and 0.99, respectively.

## Figures and Tables

**Table 1:** Mix proportion of steel fiber reinforced concrete (SFRC) and ordinary concrete (OC).

Mix No	Cement (kg/m^3^)	Water (kg/m^3^)	Sand (kg/m^3^)	Stone (kg/m^3^)	FDN-1 (kg/m^3^)	Steel Fiber Content (kg/m^3^)
MF00	341.7	164.0	661.1	1283.3	3.42	0.0
MF05	358.3	172.0	748.7	1171.0	3.58	39.3
MF05-0	358.3	172.0	748.7	1171.0	3.58	0.0
MF10	375.0	180.0	795.9	1099.1	3.75	78.6
MF10-0	375.0	180.0	795.9	1099.1	3.75	0.0
MF15	391.7	188.0	841.7	1028.7	3.92	117.9
MF15-0	391.7	188.0	841.7	1028.7	3.92	0.0
MF20	408.3	196.0	885.9	959.7	4.08	157.2
MF20-0	408.3	196.0	885.9	959.7	4.08	0.0

“05”, etc. represent 10 times the fiber content, and “-0” is the test control group specimen. For example, “MF15-0” represents the control group of V_F_ = 1.5% milling steel fiber reinforced concrete specimens. MF00 is the reference group of concrete specimens.

**Table 2:** Testing results for the fracture parameters of SFRC and ordinary concrete.

Mix No.	V_F_ (%)	P_max_ (kN)	K_IC_ MPa·m^1/2^	K_IC_ Gain Ratio	G_F_ (N·m^−1^)	G_F_ Gain Ratio	CMOD_C_ (mm)	CTOD_C_ (mm)	δ_0_ (mm)
MF00	0.0	3.1802	1.7006	-	181.410	-	0.0618	0.0324	0.0893
MF05	0.5	3.3151	2.2146	1.43	1043.8433	5.88	0.0997	0.0566	0.1203
MF05-0	0.0	2.7759	1.5493	-	177.3862	-	0.0645	0.0397	0.1068
MF10	1.0	3.6335	2.9164	1.84	1786.4485	9.86	0.1739	0.0996	0.1894
MF10-0	0.0	2.8952	1.5884	-	181.1768	-	0.0656	0.0377	0.1076
MF15	1.5	4.6427	4.3073	2.38	2331.9664	15.27	0.2638	0.1446	0.3450
MF15-0	0.0	2.9000	1.8067	-	152.7245	-	0.0789	0.0330	0.0831
MF20	2.0	6.1080	5.3880	3.45	3264.4605	20.52	0.3060	0.1692	0.3941
MF20-0	0.0	2.7034	1.5616	-	159.0965	-	0.0629	0.0326	0.0807

## Data Availability

The data presented in this study are available on request from the corresponding author.
